# An analysis of exogenous harmful substance exposure as risk factors for COPD and hypertension co-morbidity using PSM

**DOI:** 10.3389/fpubh.2024.1414768

**Published:** 2024-06-25

**Authors:** Qingyang Chen, Haodong Zhou, Jiarong Tang, Yingbiao Sun, Geriletu Ao, Hongjun Zhao, Xuhong Chang

**Affiliations:** ^1^Department of Toxicology, School of Public Health, Lanzhou University, Lanzhou, China; ^2^Department of Pulmonary and Critical Care Medicine, The Quzhou Affiliated Hospital of Wenzhou Medical University, Quzhou People's Hospital, Quzhou, China

**Keywords:** chronic obstructive pulmonary disease (COPD), hypertension, co-morbidity, propensity score matching (PSM), exogenous harmful substances, risk factors

## Abstract

**Background:**

Some occupational and environmental exposures could increase the risk of chronic obstructive pulmonary disease (COPD) and hypertension in various work and living environments. However, the effect of exposure to multiple exogenous harmful substances on COPD and hypertension co-morbidities remains unclear.

**Methods:**

Participants were selected from eight hospitals in five provinces in China using a multistage cluster sampling procedure. Participants' demographic, exposure, and disease information were collected through questionnaires, spirometry, and blood pressure examinations. Demographic data were used as matching factors, and 1:1 matching between the exposed and non-exposed groups was performed by employing propensity score matching (PSM) to minimize the influence on the results. A one-way chi-squared analysis and multifactorial logistic regression were used to analyze the association between the exposure to exogenous harmful substances (metals and their compound dust, inorganic mineral dust, organic chemicals, and livestock by-products) and the co-morbidity of COPD and hypertension.

**Results:**

There were 6,610 eligible participants in the final analysis, of whom 2,045 (30.9%) were exposed to exogenous harmful substances. The prevalence of co-morbidities of COPD and hypertension (6.0%) in the exposure group was higher than their prevalence in the total population (4.6%). After PSM, exogenous harmful substance exposure was found to be a risk factor for the co-morbidity of COPD and hypertension [odds ratio (OR) = 1.347, 95% confidence interval (CI): 1.011–1.794], which was not statistically significant before PSM (OR = 1.094, 95% CI: 0.852–1.405). Meanwhile, the results of different outcomes showed that the association between hypertension and exogenous harmful substance exposure was not statistically significant (OR = 0.965, 95% CI: 0.846–1.101). Smoking (OR = 4.702, 95% CI: 3.321–6.656), history of a respiratory disease during childhood (OR = 2.830, 95% CI: 1.600–5.006), and history of respiratory symptoms (OR = 1.897, 95% CI: 1.331–2.704) were also identified as risk factors for the co-morbidity of COPD and hypertension.

**Conclusion:**

The distribution of exogenous harmful substance exposure varies in the population, and the prevalence of co-morbidities is generally higher in susceptible populations. Exposure to exogenous harmful substances was found to be a key risk factor after adjusting for demographic confounders.

## 1 Introduction

Chronic obstructive pulmonary disease (COPD) is a common inflammatory disease of the respiratory system, characterized by persistent respiratory symptoms and restricted airflow (1, 2). In China, the life expectancy at birth was 78.2 years in 2021 (3), and currently, the population of people aged 65 years and older has reached 267 million, representing 14.2% of the total population (4). Due to the longer life expectancy and the rapid increase in the older adult population, the prevalence of COPD has been increasing in China, reaching 13.7% among people over 40 years of age. It is expected to be the seventh largest disease burden worldwide by 2030 (5), and nearly three million people die of COPD every year in the world (6). However, studies have shown that respiratory failure, the most common complication of COPD, is not the main cause of death in COPD patients. With only 4–33% of deaths in COPD patients being due to respiratory failure, the remaining 60% of deaths are caused by other co-morbidities.

Co-morbidity is defined as the presence of two or more chronic diseases in an individual at the same time (7), and people with COPD are much more likely to have multiple co-morbidities than people without COPD (8). Hypertension is the most common cardiovascular co-morbidity in COPD, with its prevalence ranging from 17 to 77% (9). Poorly controlled blood pressure can lead to diastolic dysfunction, which may be similar to symptoms associated with exercise intolerance and acute exacerbations of COPD (10). Recent studies have shown that air pollution leads to the development of oxidative stress and vascular inflammation in the body, which ultimately exacerbates the risk of illness and death in both COPD and hypertension patients (11, 12). Therefore, further research on the prevalence and risk factors of co-morbidities of COPD and hypertension is of great significance for improving people's health and reducing medical expenditures.

This study focuses on the effects of exogenous harmful substance exposure on co-morbidities of COPD and hypertension, which is different from previous studies on the effects of exogenous exposures on single COPD or hypertension. A study based on NHANES data found that co-exposure to polycyclic aromatic hydrocarbons (PAHs) was associated with COPD (OR = 1.44, 95% CI: 1.09–1.90) (13). Meanwhile, epidemiological and clinical studies have shown that heavy metals can promote the production of reactive oxygen species and induce inflammation, leading to an increased risk of hypertension, cardiac arrhythmias, and atherosclerosis (14). The effects of exogenous harmful substance exposure were diverse and complex (15), with many populations found to have been exposed to exogenous harmful substances from industries such as the chemical or smelting, textile, and animal husbandry (16). In this study, exogenous harmful substances were categorized as “metal dust, inorganic mineral dust, organic solvents, and livestock by-products.” Therefore, a study on the relationship between exposure to multiple exogenous harmful substances and the co-morbidity of COPD and hypertension could be meaningful.

Propensity score matching (PSM) has generally been used in recent studies to balance the confounding factors between groups. PSM matched the factors with large differences between groups as matching factors to reduce the influence of confounding factors and avoid errors when planning further prospective studies (17, 18). A study on a nationwide sample of US adults aged 65 years and older used PSM to adjust the population baseline data and found that influenza vaccination was associated with reducing the risk of Alzheimer's disease (RR = 0.60, 95% CI: 0.59–0.61) (19). Meanwhile, different medications and all-cause mortality in diabetic veterans were matched for demographic and clinical factors by PSM, and it was found that all-cause mortality was 0.57 times lower in patients taking metformin compared to those taking insulin or sulfonylureas (20). The above studies have shown that propensity score matching using baseline demographic data as a matching factor can fully consider the influence of factors such as age and sex on the disease itself. At the same time, it has been shown that there is an association between demographic factors and exposure to exogenous harmful substances (21, 22), and matching treatments may reduce bias during the analysis of influencing factors. Therefore, using PSM to preprocess the data makes the results of the relationship between exposure to exogenous harmful substances and the co-morbidity of COPD and hypertension more credible.

## 2 Materials and methods

### 2.1 Cohort and sample information

**Figure 1:**
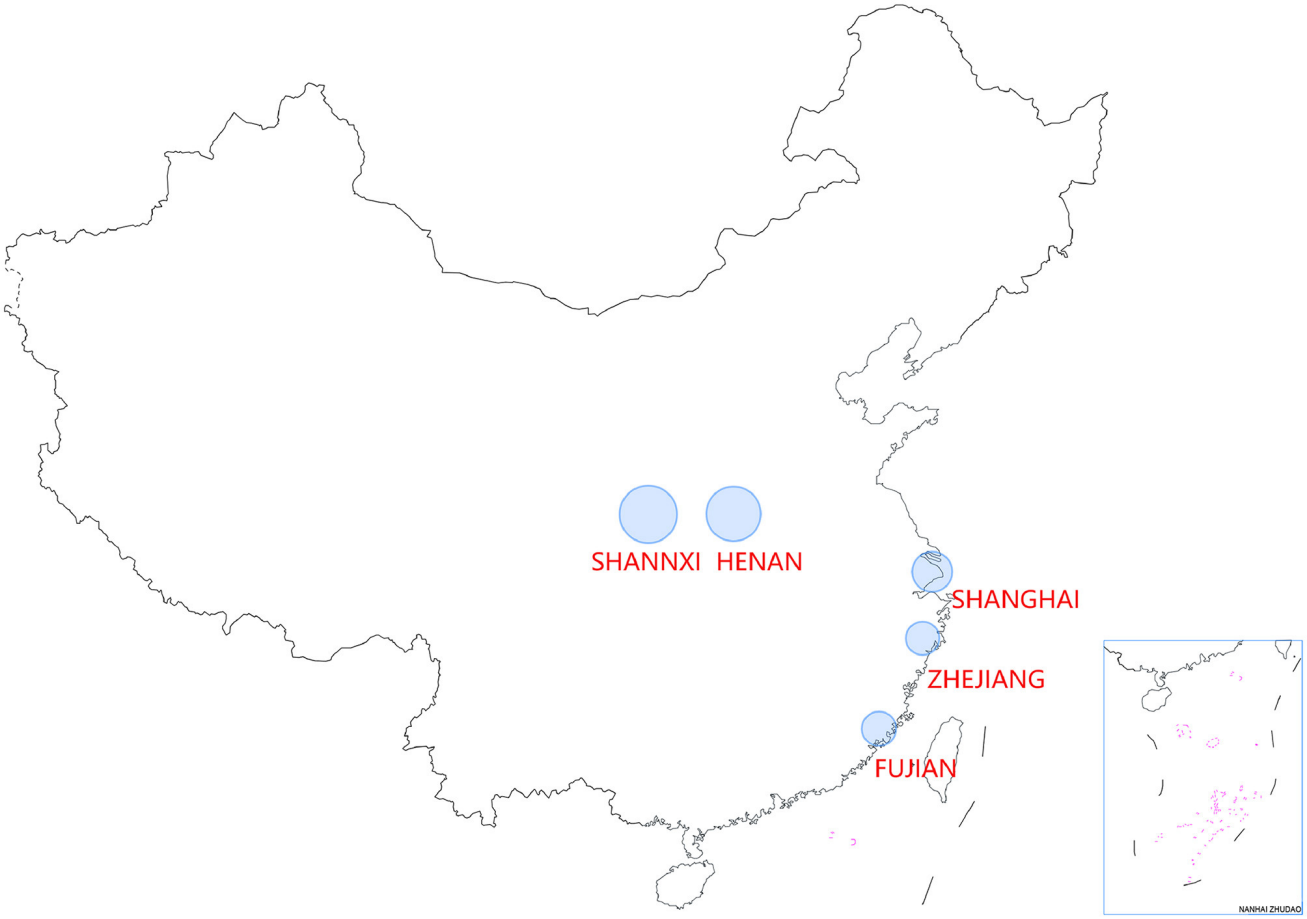
Survey points in China include the Shanghai, Shaanxi, Henan, Fujian, and Zhejiang provinces.

This is a cross-sectional study based on the COPD Pathogenesis Factors Research Cohort. The cohort was selected using a whole population sampling method from hospitals in Shanghai, Zhejiang, Fujian, Henan, and Shaanxi provinces that met the inclusion criteria (Figure 1). Participants included hospitalized patients, people who visited the hospitals for health checkups, and people who underwent community health checkups, all of whom signed informed consent forms. The inclusion criteria for the cohort population are as follows: individuals over 18 years of age; those that do not have any other underlying lung diseases as determined by chest imaging; and those who met the diagnostic criteria for COPD by pulmonary function tests, who were recognized as the diseased group, while those who did not were classified as controls. The exclusion criteria included a history of chronic lung disease other than COPD, a history of acute lung disease within the last 3 months, current malignancy with an expected survival rate of ≤ 5 years, and pregnant and lactating women, resulting in a total of 6,843 individuals being included in the cohort. This study has been reviewed by the Ethics Committee of the First Affiliated Hospital of Wenzhou Medical University (Ethics No. 2016-131). The names of the hospitals and the regional division criteria are mentioned in Table 1.

**Table 1:** Sampling sources of the study and the regional division criteria.

**Region**	**Hospital**
**Southeast**	
Zhejiang Province	First Affiliated Hospital of Wenzhou Medical University
Fujian Province	Second Affiliated Hospital of Fujian Medical University
Shanghai City	Zhongshan Hospital Affiliated to Fudan University, Central Hospital of Minhang District, Central Hospital of Feng Xian District
**Midwest**	
Henan Province	Henan Provincial People's Hospital, First Affiliated Hospital of Zhengzhou University
Shaanxi Province	Second Affiliated Hospital of Xi'an Jiao Tong University

### 2.2 Sampling and data verification

The overall sample was verified by two-person data re-entry and logical verification, and samples with obvious logical verification errors and excessive missing data were removed. Data from 6,610 cases were finally included in this study for statistical analysis, with a response rate of 96.6% and a missing rate of 3.4%, which is a sample size that meets the minimum requirement for this study. Based on Equation (23), we took *deff* = 5, α = 95%, *p* = 10.2%, *r* = 20%, and response rate = 90% to calculate the minimum sample size of this research as 3,521. We ultimately sampled 6,610 individuals in the study, which met the sample size requirement.


$$\begin{array}{l} N\;=\;deff\frac{\mu_{\alpha }^{2}p\;\left(1-p\right)}{{\;\left(pr\right)}^{2}} \end{array}$$

### 2.3 Main research content

A professional questionnaire was implemented to collect the information of the participants (24). The questionnaire included the following: (1) demographic data (age, sex, region, ethnicity, and body mass index (BMI); (2) history of exposure (biomass fuels, exogenous harmful substances, smoking, and secondhand smoke); and (3) disease history (history of a respiratory disease during childhood and history of respiratory symptoms). The exposure data were collected based on the question regarding exposure to the following substances for over 1 year, and those who selected “yes” to the following options would be recognized as having exposure to exogenous harmful substance: (1) metals and their dust such as aluminum, tin, and antimony; (2) inorganic mineral dust such as silicon dioxide, asbestos, and cement; (3) organic chemicals such as fragrances, hair dyes, and pesticides; and (4) livestock by-products such as pesticides, fertilizers, livestock manure, and animal fur/feather. The questionnaire and the spirometry and blood pressure checks were performed by trained staff from local healthcare facilities.

### 2.4 Definition and classification criteria

For the outcome indicators of this study, COPD was identified based on the following criteria: (1) chest imaging that excluded other lung diseases and (2) Forced Expiatory Volume in 1s (FEV1)/Forced Vital Capacity (FVC) ratio of < 70% after the inhalation of bronchodilators. Hypertension was defined as containing any of the following conditions: (1) systolic blood pressure >140 mmHg; (2) diastolic blood pressure >90 mmHg; and (3) previous diagnosis of hypertension and use of antihypertensive medication within 2 weeks.

As for the research factors, age categorization was based on the standard cutoff point of 65 years, and smokers were defined as those who had continually smoked for more than 6 months. Biomass fuel was defined as wood, grass, animal dung, and crop waste in this study. Exogenous harmful substances include metals and their compound dust, inorganic mineral dust, organic chemicals, and livestock by-products. History of respiratory symptoms included nasal congestion, thick yellowish-green nasal discharge, and recurrent nasal bleeding. The southeast region includes Shanghai, Zhejiang, and Fujian provinces, while the midwest region includes Henan and Shaanxi provinces.

### 2.5 Statistical analysis

PSM was used to make the case and control groups comparable in terms of demographic characteristics. Demographic characteristics such as age, sex, and ethnicity were used as matching variables; the caliper value was 0.01; and the case group was matched at a 1:1 ratio with the control group. In this study, logistic regression analysis was performed by using COPD and hypertension co-morbidity as the study endpoints, exogenous harmful substances as the independent variables, and BMI, biomass fuel exposure, smoking, exposure to secondhand smoke, history of a respiratory disease during childhood, and history of respiratory symptoms as covariates. Categorical variables were presented as real values and ratios, the chi-squared test was used for univariate comparisons, and logistic regression was used for the multivariate analysis to obtain the odds ratio (OR) and 95% confidence interval (CI) of exogenous harmful substance exposure and other factors associated with the risk of COPD combined with hypertension. We used SPSS 24.0 for statistical analysis, and the result was considered statistically significant only when the two-sided *p*-value was < 0.05.

## 3 Results

### 3.1 Participants characteristics

There were 6,610 eligible subjects, of which 2,045 (30.9%) were exposed to exogenous harmful substances. There were 3,846 (58.2%) subjects over the age of 65 years, and male subjects accounted for 59.8% of the total participants. A total of 5,423 (82.0%) subjects were from the southeast region, while only 37 (0.6%) ethnic minority participants were included. Compared with the subjects without exposure, the exposed group had significant differences in age, region, BMI, biomass fuel exposure, smoking, exposure to secondhand smoke, history of a respiratory disease during childhood, and history of respiratory symptoms (*P* < 0.05; Table 2).

**Table 2:** Distribution of baseline characteristics of subjects for different exposure scenarios (%).

**Variables**	**Total *n* = 6,610**	**Exposed *n* = 2,045**	**No*n*-exposed *n* = 4,565**	**χ^2^**	***P***
Age (year)				128.49	**< 0.001**
< 65	2,764 (41.8)	645 (31.5)	2,119 (46.4)		
≥65	3,846 (58.2)	1,400 (68.5)	2,446 (53.6)		
Sex				1.56	0.212
Male	3,953 (59.8)	1,246 (60.9)	2,707 (59.3)		
Female	2,657 (40.2)	799 (39.1)	1,858 (40.7)		
Region				305.76	**< 0.001**
Southeast	5,423 (82.0)	1,930 (94.4)	3,493 (76.5)		
Midwest	1,187 (18.0)	115 (5.6)	1,072 (23.5)		
Ethnicity				2.64	0.104
Han	6,573 (99.4)	2,029 (99.2)	4,644 (99.5)		
Others	37 (0.6)	16 (0.8)	21 (0.5)		
BMI (kg/m^2^)				14.10	**0.001**
≤ 18.5	214 (3.2)	83 (4.1)	131 (2.9)		
18.5–24	2,995 (45.3)	970 (47.4)	2,025 (44.4)		
>24	3,401 (51.5)	992 (48.5)	2,409 (52.8)		
Biomass fuel exposure				38.06	**< 0.001**
Yes	318 (4.8)	148 (7.2)	170 (3.7)		
No	6,292 (95.2)	1,897 (92.8)	4,395 (96.3)		
Smoking				29.00	**< 0.001**
Yes	4,431 (67.0)	1,600 (35.0)	2,965 (65.0)		
No	2,179 (33.0)	579 (28.3)	1,466 (71.7)		
Secondhand smoke				125.28	**< 0.001**
Yes	2,619 (39.6)	1,016 (49.7)	1,603 (35.1)		
No	3,991 (60.4)	1,029 (50.3)	2,962 (64.9)		
History of a respiratory disease during childhood				5.61	**0.018**
Yes	216 (3.3)	51 (2.5)	165 (3.6)		
No	6,394 (96.7)	2,994 (97.5)	4,400 (96.4)		
History of respiratory symptoms				22.87	**< 0.001**
Yes	654 (9.9)	256 (12.5)	398 (8.7)		
No	5,956 (90.1)	1,789 (87.5)	4,167 (91.3)		

The bold values indicates statistically significant (*P* < 0.05).

### 3.2 Prevalence of COPD and hypertension co-morbidities in total group and subgroups

This study aimed to clarify the overall prevalence of co-morbidities of COPD and hypertension and the effect of exogenous harmful substance exposure on this prevalence. It used the stratified one-way chi-squared analysis to discuss the effect of exposure factors on the prevalence in each subgroup. The results showed that the prevalence rates of COPD, hypertension, and co-morbidity of COPD and hypertension were 16% (1,097/6,610), 32% (2,116/6,610), and 4.6% (306/6,610), respectively. There was a significant difference in single disease prevalence and co-morbidity prevalence between participants who were exposed to exogenous harmful substances (21.5, 34.1, and 6.0%) and those who were not exposed (14.4, 31.1, and 4.0%). Furthermore, there was also a significant difference in co-morbidity prevalence in subgroups based on sex, ethnicity, smoking, secondhand smoke exposure, history of a respiratory disease during childhood, and history of respiratory symptoms (*P* < 0.05; Table 3).

**Table 3:** Prevalence in different exposure situations and subgroups.

**Variables**	**Prevalence in total**		**Prevalence in the exposed group**		**Prevalence in the non-exposed group**		***P***
	**Case/total**	**Rate (%)**	**Case/total**	**Rate (%)**	**Case/total**	**Rate (%)**	
COPD	1,097/6,610	16.6	440/2,045	21.5	657/4,565	14.4	**< 0.001**
Hypertension	2,116/6,610	32.0	698/2,045	34.1	1,418/4,565	31.1	0.013
Co-morbidities	**306/6,610**	**4.6**	**122/2,045**	**6.0**	**184/4,565**	**4.0**	**0.001**
**Age (year)**							
< 65	46/2,764	1.7	13/645	2.0	33/2,119	1.6	0.428
≥65	260/3,846	6.8	109/1,400	7.8	151/2,446	6.2	0.054
*P*		**< 0.001**		**< 0.001**		**< 0.001**	
**Sex**							
Male	256/3,953	6.5	105/1,246	8.4	151/2,707	5.6	**0.001**
Female	50/2,657	1.9	17/799	2.1	33/1,858	1.8	0.541
*P*		**< 0.001**		**< 0.001**		**< 0.001**	
**Region**							
Southeast	271/5,423	5.0	113/1,930	5.9	158/3,493	4.5	**0.031**
Midwest	35/1,187	2.9	9/115	7.8	26/1,072	2.4	**0.001**
*P*		**0.002**		0.386		**0.002**	
**Ethnicity**							
Han	305/6,573	4.6	122/2,029	6.0	183/4,544	4.0	**< 0.001**
Others	1/37	2.7	0/16	0.0	1/21	4.8	0.376
*P*		0.576		0.312		0.864	
**BMI (kg/m** ^2^ **)**							
≤ 18.5	9/214	4.2	6/83	7.2	3/131	2.3	0.079
18.5–24	141/2,995	4.7	60/970	6.2	81/2,025	4.0	**0.008**
>24	156/3,401	4.6	56/992	5.6	100/2,409	4.2	0.058
*P*		0.931		0.778		0.571	
**Biomass fuel exposure**							
Yes	18/318	5.7	15/148	10.1	3/170	1.8	**0.001**
No	288/6,292	4.6	107/1,897	5.6	181/4,395	4.1	**0.008**
*P*		0.370		**0.026**		**0.126**	
**Smoking**							
Yes	259/4,431	5.8	107/1,466	7.3	152/2,965	5.1	**0.007**
No	47/2,179	2.2	15/579	2.6	32/1,600	2.0	0.145
*P*		**< 0.001**		**< 0.001**		**< 0.001**	
**Secondhand smoke**							
Yes	120/2,619	4.6	64/1,016	6.3	56/1,603	3.5	**0.001**
No	186/3,991	4.7	58/1,029	5.6	128/2,962	4.3	0.085
*P*		0.882		0.527		0.175	
**History of a respiratory disease during childhood**							
Yes	24/216	11.1	7/51	13.7	17/165	10.3	0.497
No	282/6,394	4.4	115/1,994	5.8	167/4,400	3.8	**< 0.001**
*P*		**< 0.001**		**0.018**		**< 0.001**	
**History of respiratory symptoms**							
Yes	56/654	8.6	25/256	9.8	31/398	7.8	**0.002**
No	250/5,956	4.2	97/1,789	5.4	153/4,167	3.7	0.378
*P*		**< 0.001**		**0.006**		**< 0.001**	

The bold values indicates statistically significant (*P* < 0.05).

### 3.3 Using PSM to balance the demographic baseline between groups

**Figure 2:**
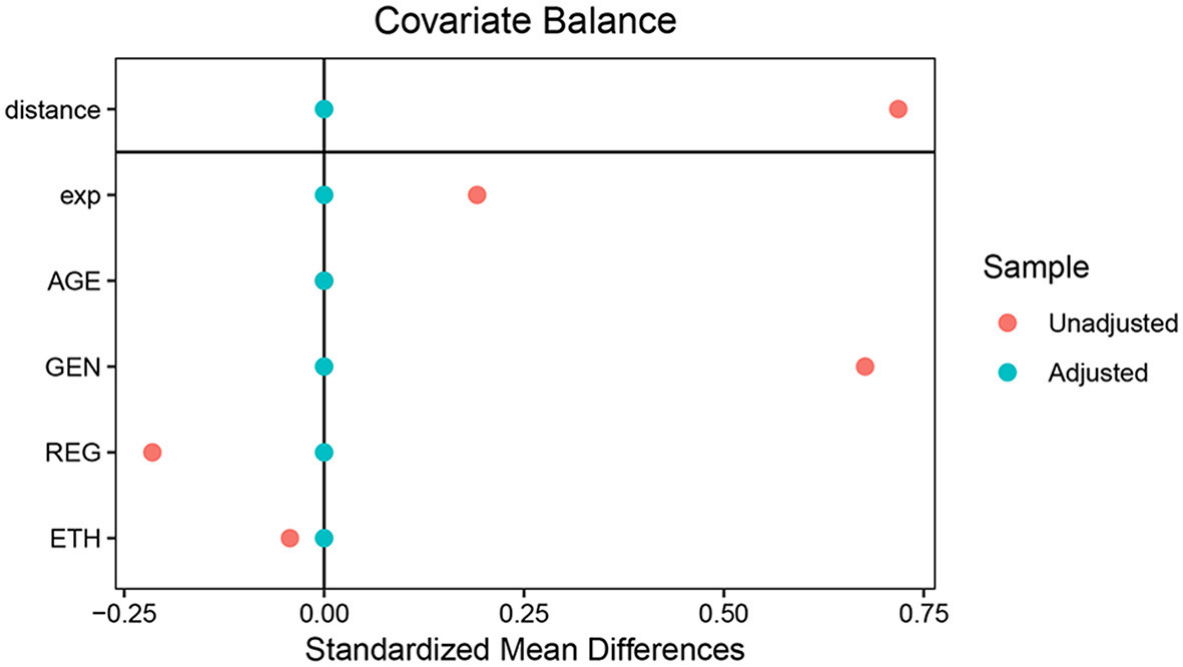
Distribution of demographic characteristics of the two groups before and after matching.

Based on the differences in participants' characteristics, this study used PSM to preprocess the data to eliminate these differences. Non-intervention variables such as age, sex, region, and ethnicity were selected as matching factors for PSM, and each group contained 2,041 participants after being matched at a ratio of 1:1 with a caliper value of 0.02. After matching, the differences in the above factors were found to no longer be statistically significant between the two groups (*P* > 0.05; Figure 2).

### 3.4 Effects of exogenous harmful substance exposure and other risk factors

After PSM, the univariate analysis was used to analyze the distribution of other influencing factors between the case group and the control group. The results showed that there were different distributions between groups in variables such as biomass fuel exposure, exogenous harmful substance exposure, smoking, history of a respiratory disease during childhood, and history of respiratory symptoms (*P* < 0.05; Table 4).

**Table 4:** Univariate analysis of influencing factors after matching (%).

**Variables**	**Case group**	**Control group**	**χ^2^**	***P***
BMI (kg/m^2^)			0.38	0.827
≤ 18.5	9 (4.3)	144 (3.7)		
18.5–24	101 (47.9)	1,801 (46.5)		
≥24	101 (47.9)	1,926 (49.8)		
Biomass fuel exposure			4.59	**0.032**
Yes	16 (7.6)	171 (4.4)		
No	195 (92.4)	3,700 (95.6)		
Exposure to exogenous harmful substances			5.44	**0.020**
Yes	122 (57.8)	1,919 (49.6)		
No	89 (42.2)	1,952 (50.4)		
Smoking			93.61	**< 0.001**
Yes	169 (80.1)	1,778 (45.9)		
No	42 (19.9)	2,093 (54.1)		
Secondhand smoke			0.10	0.753
Yes	93 (44.1)	1,749 (45.2)		
No	118 (55.9)	2,122 (54.8)		
History of a respiratory disease during childhood			20.66	**< 0.001**
Yes	16 (7.6)	93 (2.4)		
No	195 (92.4)	3,778 (97.6)		
History of respiratory symptoms			22.36	**< 0.001**
Yes	45 (21.3)	416 (10.7)		
No	166 (78.7)	3,455 (89.3)		

The bold values indicates statistically significant (*P* < 0.05).

To clarify the role of exogenous harmful substance exposure in the co-morbidities of COPD and hypertension and to assess the impact of the PSM data processing method on the results, binary logistic regression analyses were used in this study to regress the factors before and after PSM (Table 5). The results showed that exposure to multiple exogenous harmful substances (OR = 1.332, 95% CI: 1.087–1.631) became statistically significant after matching. This finding indicated that the application of PSM successfully reduced the influence of demographic factors on the data analysis and made the results statistically significant. Smoking, history of a respiratory disease during childhood, and history of respiratory symptoms were also found to be the risk factors for the co-morbidities of COPD and hypertension (*P* < 0.05).

**Table 5:** Logistic regression analysis of risk factors for co-morbidities of COPD and hypertension.

**Variables**	**OR value**	***P***	**OR value**	***P***
	**Before PSM (95% CI)**		**After PSM (95% CI)**	
Underweight	0.814 (0.405, 1.637)	0.564	1.006 (0.491, 2.060)	0.987
Overweight/obesity	0.970 (0.765, 1.230)	0.802	1.017 (0.762, 1.358)	0.909
Biomass fuel exposure	0.997 (0.605, 1.642)	0.990	1.384 (0.799, 2.396)	0.246
Exposure to exogenous harmful substances	**1.094 (0.952, 1.516)**	**0.349**	**1.347 (1.011, 1.794)**	**0.042**
Smoking	3.971 (3.025, 5.214)	< 0.001	4.702 (3.321, 6.656)	< 0.001
Secondhand smoke	0.854 (0.670, 1.089)	0.204	0.747 (0.561, 0.996)	0.047
History of a respiratory disease during childhood	2.505 (1.592, 3.942)	< 0.001	2.830 (1.600, 5.006)	< 0.001
History of respiratory symptoms	1.849 (1.357, 2.520)	< 0.001	1.897 (1.331, 2.704)	< 0.001

The bold values indicates statistically significant (*P* < 0.05).

### 3.5 The role of exogenous harmful substance exposure in different conditions

Based on the matched data, this study performed logistic regression analyses of the three types of study outcomes: COPD, hypertension, and co-morbidities, with the inclusion of all explanatory variables and harmonic commands to clarify the relationship between exogenous harmful substances and co-morbidities (Table 6). The results showed that exogenous harmful substances increased the risk of COPD (OR = 1.348, 95% CI: 1.130–1.609), but the association between exogenous harmful substances and hypertension was not statistically significant (OR = 0.965, 95% CI: 0.846–1.101). Based on these results, we can conclude that exogenous harmful substances are the only risk factors for the co-morbidities of COPD and hypertension (*P* < 0.05).

**Table 6:** Relationship between exogenous harmful substances and diseases.

**Type of disease**	**OR value**	***P***
COPD	1.348 (1.130, 1.609)	0.001
Hypertension	0.965 (0.846, 1.101)	0.596
Co-morbidities of COPD and hypertension	1.347 (1.011, 1.794)	0.042

## 4 Discussion

To the best of our knowledge, the distribution of exposure to multiple exogenous harmful substances varies across populations with different baseline characteristics. The overall prevalence of co-morbidity between COPD and hypertension was 4.6%, and 27.9% (306/1,097) of the COPD patients had co-morbid hypertension in this study. In addition, the prevalence of co-morbidity between COPD and hypertension was higher in the exposed group than in the non-exposed group, which was also statistically significant in many subgroups. After PSM and logistic regression analyses, we found that exposure to exogenous harmful substances increased the risk of COPD and hypertension co-morbidity, while this finding was not statistically significant in the logistic regression analysis before PSM.

We found that subjects of older age, exposed to tobacco, and with a history of respiratory symptoms have a higher risk of exposure to exogenous harmful substances, which may provide a theoretical basis for screening high-risk groups. In addition, our study found a correlation between having a history of respiratory symptoms and exposure to exogenous harmful substances. Lung tissue in older adults is more susceptible to harmful particulate matters due to a progressive decline in physical function and the immune system (21). Meanwhile, COPD patients with a combined exposure to environmental factors and smoking accounted for 58.8% of all smoking patients in southern China (25). It is worth noting that this study also found that those with pre-existing respiratory symptoms had a higher rate of exposure to exogenous harmful substances, which may be due to the protective barrier of the respiratory system being chronically impaired (26) so people became more susceptible to exogenous harmful substances. Therefore, we posit that the demographic characterization of exposure to multiple exogenous harmful substances may assist in early screening for disease and risk factors for COPD and hypertension co-morbidity.

This study showed that subgroups with a history of tobacco exposure, a history of a respiratory disease in childhood, and a history of respiratory symptoms in the exposed group have a higher prevalence of co-morbidity between COPD and hypertension. Both active and passive smoking, as known risk factors for cardiovascular and respiratory diseases (27, 28), also demonstrated a positive association with the prevalence of COPD and hypertension co-morbidity in this study. Meanwhile, a history of respiratory diseases such as pneumonia or bronchitis in childhood can lead to irreversible damage in the airway passage, thereby increasing the probability of developing respiratory diseases in adulthood (29). Similarly, a history of respiratory disease in childhood is positively associated with the mass of left ventricular masses in the heart during adulthood, and the probability of developing left ventricular masses in the heart is much higher in hypertensive people than in the general population (30). Furthermore, having a history of respiratory symptoms was also found to be correlated with a higher prevalence of COPD and hypertension co-morbidity, which confirms this study's present characterization of the exposure profile. The presence of pre-existing respiratory symptoms can indicate an immunocompromised immune system, which not only makes them more susceptible to exposure to harmful substances but also makes them more prone to COPD and high blood pressure (31, 32). Thus, the results of the increasing prevalence of COPD and hypertension co-morbidity in susceptible populations indicate that we should pay more attention to susceptible populations to raise their public health awareness.

In this study, the propensity score was combined with traditional regression analysis to eliminate the differences in some variables between the two groups, which could better explain the relationship between exogenous harmful substances and the co-morbidity of COPD and hypertension. Previous studies using PSM have found that salvage radiotherapy significantly improves the oncologic outcomes of patients after radioprostatectomy (33). In addition, the regression results of this study showed that exposure to exogenous harmful substances is a risk factor for COPD and hypertension co-morbidity only after PSM. There have been many studies indicating that demographic factors, such as sex and age, are associated with both COPD and hypertension. Older individuals and male subjects have been found to be more likely to be exposed to exogenous harmful substances (21, 22). Therefore, the relationship between exogenous harmful substances and study outcomes in unmatched regression analyses may have been influenced by the combination of demographic factors with the independent and outcome variables and thus shown to be not statistically significant. When the basic characteristics of the experimental and control groups are not comparable, the results may reflect the differences in the baseline characteristics rather than the outcomes caused by different exposures (34). In this study, an unbiased result was obtained by using PSM, which provided a more accurate estimate of likely causality. However, it should be noted that the sample size of the data was reduced after using PSM, which suggests that we should strictly review the PSM process in subsequent studies to avoid misleading results.

Based on the logistic regression results of this study, exposure to exogenous harmful substances was found to be a key factor in increasing the risk of COPD and hypertension co-morbidity. A Korean COPD cohort study showed that occupational exposure to harmful chemicals and grain dust caused airway inflammation and led to substantial airway destruction and remodeling, resulting in a 1.714 times higher risk of COPD than those without an occupational exposure history (35). Meanwhile, the acute exacerbation rate of COPD in the area within 500 m of the livestock farm was higher than that in the control area due to the high levels of pathogens such as bacteria, viruses, and endotoxins emitted in the environment [Incidence Rate Ratio (IRR) = 1.28, 95% CI: 1.06–1.55] (36, 37). Hypertension can also be affected by heavy metals since recent studies have shown that blood lead levels (BLL) are a primary indicator for assessing lead exposure in human populations, and an increase in BLL may lead to hypertension and poor lung function (38). Therefore, we should continue to explore the risky role of exogenous harmful substances in the co-morbidity of COPD and hypertension, and the introduction of such exposures into early screening processes for the disease could help reduce the incidence of COPD co-morbidities and potentially lower mortality rates in COPD patients.

To clarify the true role of exogenous harmful substance exposure on the co-morbidity of COPD and hypertension, we performed additional multifactorial regression analyses with different outcomes. The results showed that exogenous harmful substance exposure was a risk factor for COPD and the co-morbidity of COPD and hypertension, but the association between hypertension alone was not statistically significant. Previous studies have shown an association between heavy metal exposure and hypertension development (14), but essentially no studies have reported an association between exposures to substances such as inorganic mineral dust and agricultural and livestock by-products and hypertension. The results demonstrate that exposure to exogenous harmful substances contributes to the development of COPD and that the exposure increases the risk of hypertension co-morbidity in those who already have COPD. However, since the data in our study only included outcome variables with COPD and hypertension, we were unable to determine whether the results directly led to an increased risk of COPD and hypertension co-morbidity or an increased risk of all kinds of COPD morbidity. We intend to be mindful in the future data collection and study design to ascertain the actual association between exogenous harmful substances and the co-morbidity of COPD and hypertension.

This study also included proven risk factors for COPD, such as biomass fuel exposure (39) and smoking (40), where tobacco exposure was found to increase the risk of co-morbidities of COPD and hypertension. However, the association between biofuel exposure and co-morbidity was not statistically significant, which may be due to the use of biomass fuel being closely linked to factors such as sex and region (41, 42). Some studies have shown that ethnic minority populations in the central and western regions use more straw and firewood for daily cooking and heating due to the gap between their economic and educational levels (43). In this study, PSM was used to match the regional and ethnic differences between the exposed and non-exposed groups of exogenous harmful substances, which resulted in no statistical significance in the regression analyses.

Furthermore, this study found that having a history of a respiratory disease in childhood and a history of respiratory symptoms are risk factors for COPD and hypertension co-morbidity. Previous studies suggested that susceptibility to multiple and prolonged episodes of childhood bronchiolitis may lead to airway compromise, and the subsequent airway stenosis that occurs during airway remodeling may impair normal airway development, thereby leading to an increased incidence of lung disease in adulthood (44). At the same time, childhood asthma has been found to elevate aorta-femoral pulse wave velocity (AF-PWV) in adulthood, which serves as a functional marker of arterial vulnerability and is considered to be caused by organ damage, such as elevated blood pressure (45). Although we found that people with a history of previous respiratory symptoms were more likely to be exposed to exogenous harmful substances and had a higher prevalence of COPD and hypertension co-morbidities, the causal relationship between symptom history and disease onset is uncertain. Therefore, further prospective studies are necessary to explore this finding.

## 5 Conclusion

Hypertension, being the main co-morbid condition associated with COPD, has a large negative impact on the treatment and prognosis. We found that the distribution of exposure to multiple exogenous harmful substances varies in the population, and the prevalence of COPD and hypertension co-morbidities is generally higher in susceptible populations. Exposure to multiple exogenous harmful substances was found to be a key risk factor for COPD and hypertension co-morbidity after PSM. Therefore, clarifying the relationship between multiple exogenous harmful substance exposures and COPD and hypertension co-morbidities is important for preventing and controlling the occurrence of COPD co-morbidities and reducing mortality.

## Data availability statement

The raw data supporting the conclusions of this article will be made available by the authors, without undue reservation.

## Ethics statement

The studies involving humans were approved by Ethics Committee of the First Affiliated Hospital of Wenzhou Medical University (Ethics No. 2016-131). The studies were conducted in accordance with the local legislation and institutional requirements. The participants provided their written informed consent to participate in this study.

## Author contributions

QC: Data curation, Investigation, Methodology, Software, Visualization, Writing – original draft. HZho: Data curation, Software, Writing – review & editing. JT: Data curation, Formal analysis, Software, Writing – review & editing. YS: Investigation, Project administration, Resources, Supervision, Writing – review & editing. GA: Project administration, Resources, Writing – review & editing. HZha: Conceptualization, Funding acquisition, Methodology, Project administration, Resources, Supervision, Validation, Writing – review & editing. XC: Conceptualization, Funding acquisition, Methodology, Project administration, Resources, Supervision, Validation, Writing – review & editing.
